# Antimicrobial effectiveness of aqueous and alcoholic herbal extracts on *Streptococcus mutans*: A systematic review and meta-analysis of randomized controlled trials

**DOI:** 10.22038/AJP.2024.24314

**Published:** 2025

**Authors:** Kosar Ghandehari, Marzie Boskabadi, Alireza Sarraf Shirazi, Masoumeh Sadeghi, Taraneh Movahhed

**Affiliations:** 1 *Faculty of Dentistry, Mashhad University of Medical Sciences, Mashhad, Iran*; 2 *Department of Pediatric Dentistry, Faculty of Dentistry, Mashhad University of Medical Sciences, Mashhad, Iran*; 3 *Department of Epidemiology, Faculty of Health, Mashhad University of Medical Sciences, Mashhad, Iran*

**Keywords:** Streptococcus mutans, Herbal extract, Aqueous extract, Alcoholic extract, Dental caries

## Abstract

**Objective:**

Debates about the efficacy of herbal products in oral care have inspired researchers to conduct a large number of trials. This systematic review and meta-analysis aimed to assess the antibacterial efficacy of aqueous and alcoholic herbal extracts against *Streptococcus mutans* (SM) as the main cariogenic microorganism.

**Materials and Methods:**

Online databases PubMed, Scopus, Cochrane Library, Web of Science, and Magiran were searched for randomized controlled trials evaluating the efficacy of herbal products against SM published up to July 2023. Meta-analyses were performed for immediate and long-term effects based on random effect model.

**Results:**

Out of 57 studies that met the selection criteria for systematic review, 26 were subjected to meta-analysis. Considering both immediate and long-term effects on salivary SM, aqueous and alcoholic herbal extracts were significantly superior in the reduction of SM over non-herbal agents (immediate: SMD = -1.16; 95%CI: -2.03, -0.29, long-term: SMD = -0.76; 95%CI: -1.35, -0.17). However, the difference was not significant in the reduction of SM of plaque (SMD = -0.30, 95%CI: -1.25, 0.65). The subgroup analyses showed no significant difference compared to chlorhexidine (p>0.05). The overall quality of evidence was considered low based on GRADE tool.

**Conclusion:**

Current evidence suggests encouraging results for herbal extracts in reduction of SM, but not over chlorhexidine. However, there is still insufficient evidence to recommend them as the first option for oral care. High-quality randomized controlled trials are required to assert the safety and effectiveness of them for preventing dental caries.

## Introduction

Dental caries are prevalent infectious diseases and a global public health concern interfering with quality of life (Ganapathi and Prabakar, 2019; Kazeminia et al., 2020; Mathur and Dhillon, 2018; Selwitz et al., 2007). About 60 to 90 percent of children and almost all adults are struggling with its complications such as sleep deprivation, malnutrition, and loss of productivity at school and work, and they produce poor aesthetic and psychological impact (Kale et al., 2020; Macfarlane et al., 2002; Peres et al., 2019). Regarding the high cost of operative treatments as well as lack or insufficiency of health insurance coverage and the possibility of recurrence, non-operative preventive treatments are gaining more attention (Chandrashekar et al., 2019b; Selwitz et al., 2007).

There is evidence that oral microbiome plays a key role in the etiology of dental caries (Usha and R, 2009) and the bacterial growth can be prevented through application of a combination of mechanical removal of the biofilm and chemical methods (Barnett, 2006; Figuero et al., 2019; Osso and Kanani, 2013; Van Der Weijden and Hioe, 2005). Mechanical methods are the main and most common means of plaque control, while chemical methods play an adjuvant role (Dentino et al., 2005; Toshniwal et al., 2022). Currently, chemical products (e.g. chlorhexidine and cetylpyridinium chloride) are commonly used as antimicrobial agents (Chen et al., 2013). Nonetheless, owing to some adverse effects (e.g. tooth staining, altered taste, supra-gingival calculus accumulation, oral pathogens resistance and changes in microbial flora), alternative approaches such as application of medicinal plants could be considered (Chen et al., 2013; Homoki et al., 2018; Khoramian Tusi et al., 2020; Usha and R, 2009; Yadav et al., 2017). Nearly 80% of the global population (with a tendency to low socioeconomic communities) consume medicinal plants for primary health care (Farnsworth et al., 1985; Saxena et al., 2017), including dental care. Medicinal plants favor low toxicity, good biocompatibility, and being available and affordable, resulting in gaining rising attention worldwide (Abubakar and Haque, 2020; Jain et al., 2013; Saxena et al., 2017). This has inspired numerous trials evaluating their efficacy in oral biofilm inhibition. To the best of the authors’ knowledge, there is a debate in the literature over effectiveness of aqueous and alcoholic herbal extracts on the reduction of oral *Streptococcus mutans* (SM) as the main cariogenic microorganism.

The present systematic review and meta-analysis aim to analyze the best available evidence on the efficacy of medicinal plants to help researchers, policymakers, and stakeholders learn about the gaps in oral health studies in order to implement necessary measures towards improving oral health worldwide, particularly in less privileged regions.

## Materials and Methods

The present study was conducted in accordance with Preferred Reporting Items for Systematic Reviews and Meta-Analyses (PRISMA) guidelines (Page et al., 2021). The study protocol was registered on PROSPERO (registration No. CRD42022293208). The research question was whether aqueous and alcoholic herbal extracts are effective in reduction of oral SM level, with the following PICO: Population: children and adults irrespective to age and sex; Intervention: oral care products containing aqueous, alcoholic or hydro-alcoholic herbal extracts (regardless of concentration); Comparison: conventional chemical methods or placebo; and Outcome: reduction in SM count of saliva or plaque.

**Search strategy and eligibility criteria**

We performed a systematic search of online literature databases including PubMed, the Cochrane Library, Scopus, Web of Science, and Magiran from inception to July 2023 with no language restriction. We also did a manual search on the bibliography of the retrieved articles and previous relevant reviews to minimize missing related studies. The search conducted on databases is illustrated on Table 1.

All retrieved literature was uploaded into EndNote version 8 and duplicates were removed. Two authors (KG and TM) screened articles independently for studies meeting our selection criteria by reviewing the titles and abstracts. Inclusion criteria included randomized controlled trials (RCTs) studying the efficacy of aqueous and alcoholic herbal extracts in controlling oral SM level and eventually, preventing dental caries. No limits were applied to the year of study or language. We excluded non-research articles, or studies of participants with mental or physical disability or any systemic disease that could disturb the result or studies that included individuals with a history of recent (within the previous three month) antibiotic intake.

In the second phase, two authors (KG and MB) reviewed the full texts of the remaining articles fulfilling all the selection criteria and having the data for analysis. A third author (AS) interfered if any disagreement appeared. In case of potential suitability of the study and lack of sufficient data, we made two attempts to contact corresponding authors via e-mail asking for missing data.

**Data extraction**

Data of country or origin, publication year, demographic characteristics of patients, sample size, study duration, intervention and control, methods of sample collection, bacterial counting and study outcome were gathered from eligible studies. Data graph digitizer was used for figure analysis. The following outcomes were collected from both intervention and control groups:

-SM count before and after the oral care product consumption;

-Immediate effects: studies in which the sample was acquired within two hours of a single dose consumption of oral care product;

-Long-term effects: studies with repeated regular consumption of oral care product.

**Assessment of study quality and credibility of meta-analysis**

Risk of bias assessment was implemented through Revised Cochrane risk-of-bias tool for randomized trials (ROB2) within five aspects (appraising randomization process, assignment to intervention, adhering to intervention, missing outcome data, measurement of outcome and selection of reported results) (Higgins et al., 2019). To assess the quality of evidence, we used Grading of Recommendation Assessment, Development and Evaluation (GRADE) (Schünemann et al., 2019).

**Data analysis**

Various dilution of samples for bacterial counting resulted in different scale of measurements. Thus, we used the Standardized Mean Difference (SMD) model to pool the effect size for quantitative synthesis of data (Borenstein et al., 2021). Funnel plot helped to detect publication bias in the presence of at least 10 studies (Page et al., 2019; Sterne et al., 2011). An I^2 ^statistic of>30% was suggestive of heterogeneity. Sensitivity analysis displayed the extent of which a particular study influences the overall result. A two-tailed p-value<0.05 was considered statically significant. Subgroups were determined according to the control groups being defined as either placebo, chlorhexidine, or other anti-plaque products. We analyzed data using STATA software version 14 (Stata corp., College Station, TX, USA).

## Results

**Search results**

**Figure 1:**
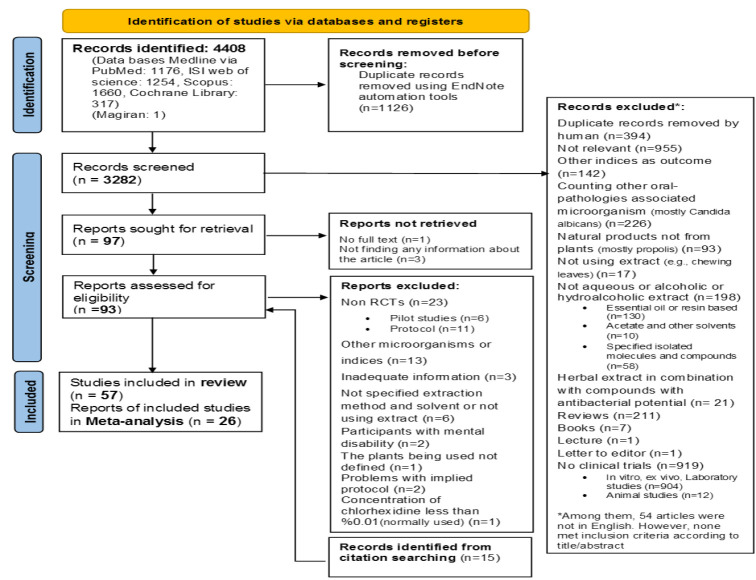
PRISMA flowchart of the present review article

The initial search identified 4408 records. Following duplication removal, 3282 articles remained for title and abstract screening; out of 3282 articles, 3185 were excluded with reasons provided in Figure 1. 54 of these excluded studies were in languages other than English or Persian, and were removed since they did not meet inclusion criteria according to their title and abstract. The full text of the remaining articles was retrieved for further assessment. Of these studies, 55 did not meet the selection criteria. Finally, out of 57 articles that met the inclusion criteria, 26 had adequate data for meta-analysis (Figure 1). The characteristics of these 57 included studies are summarized in Table 2. 

**Characteristics of the included studies**

The sample size ranged from 20 to 1434 with a total of 4333 participants being enrolled. Among the included studies, 31 trials were performed on children (about 75% of participants), 45 studies reported long-term effect and 18 studies reported immediate effect. The trial duration varied from 3 days to 9 months in long-term examination. Salivary samples were collected in 49 studies (stimulated saliva in 10 studies and unstimulated in 27 studies), whereas plaque samples were examined in 8 studies. *Glycyrrhiza glabra* (liquorice) (Almaz et al., 2017; Helmy et al., 2021; Jain et al., 2013; Kumar et al., 2020; Oznurhan et al., 2019; Pathi et al., 2021), *Punica granatum* (Mishra et al., 2019; Nobrega et al., 2015; Pinni et al., 2018; Singla et al., 2018; Srilekha and Prabakar, 2018; Umar et al., 2016), *Salvadora persica* (Al-Dabbagh et al., 2016; Bhat et al., 2012; Jauhari et al., 2015; Khalessi et al., 2004; Siddeshappa et al., 2018; Srilekha and Prabakar, 2018) and *Terminalia chebula* (Megalaa et al., 2018; Mishra et al., 2019; Nayak et al., 2012; Nayak et al., 2010; Velmurugan et al., 2013) were the most common sources of herbal extracts within the included studies. Overall, 32 articles used chlorhexidine (as the gold standard for antibacterial oral care) for control groups. The majority of trials were conducted in India and Iran (35 and 6 trials, respectively).

**Data synthesis**

For meta-analysis, 26 studies (including 487 Adults and 926 children) were considered (Table 2). Considering the variety of consumption duration and the origin of samples, the retrieved articles were analyzed within three groups:

**Immediate effect on salivary SM**:

Seven trials had data related to immediate effect of herbal products on oral SM (Bhat et al., 2012; Helmy et al., 2021; Jain et al., 2013; Jain and Jain, 2016; Megalaa et al., 2018; Nayak et al., 2012; Pathi et al., 2021). The studies had a high heterogeneity (I^2^=94.6%). The sensitivity analysis did not exclude any of these studies.

**Figure 2:**
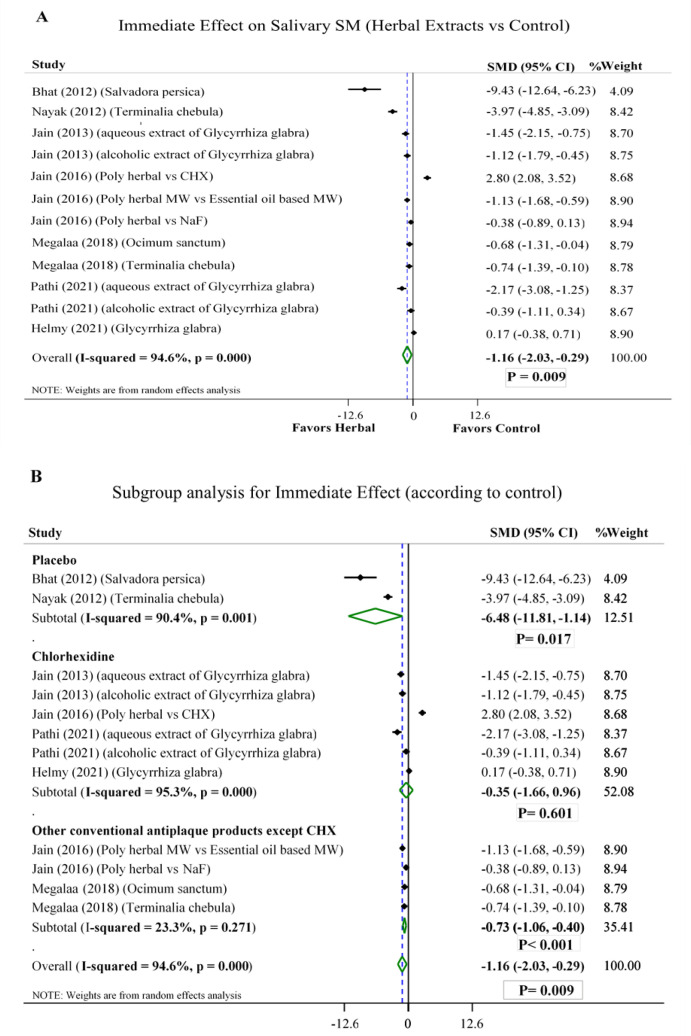
Forest plot of comparison: aqueous and alcoholic herbal extracts vs A) all controls B) subgroups (placebo, chlorhexidine, other conventional antiplaque products); SM= Streptococcus mutans; CHX= Chlorhexidine; NaF= sodium fluoride; MW= mouthwash

 Dental care products containing aqueous and alcoholic herbal extracts were significantly more likely to eliminate salivary SM compared to non-herbal ones [SMD -1.16, 95% CI (-2.03 to -0.29)] (Figure 2a). However, the subgroup analysis did not favor herbal products compared to chlorhexidine [SMD -0.35, 95% CI (-1.66 to 0.96)] (Figure 2b). 

Out of four RCTs that used chlorhexidine (Helmy et al., 2021; Jain et al., 2013; Jain and Jain, 2016; Pathi et al., 2021), 3 RCTs applied liquorice extract for intervention. Two trials noticed superiority of liquorice in children (Jain et al., 2013; Pathi et al., 2021) and one trial favored chlorhexidine in adults (Jain and Jain, 2016). Compared to sodium fluoride, two studies observed favorable reduction of SM for herbal extracts (Jain and Jain, 2016; Megalaa et al., 2018).

**Long-term effect on salivary SM:**

**Figure 3:**
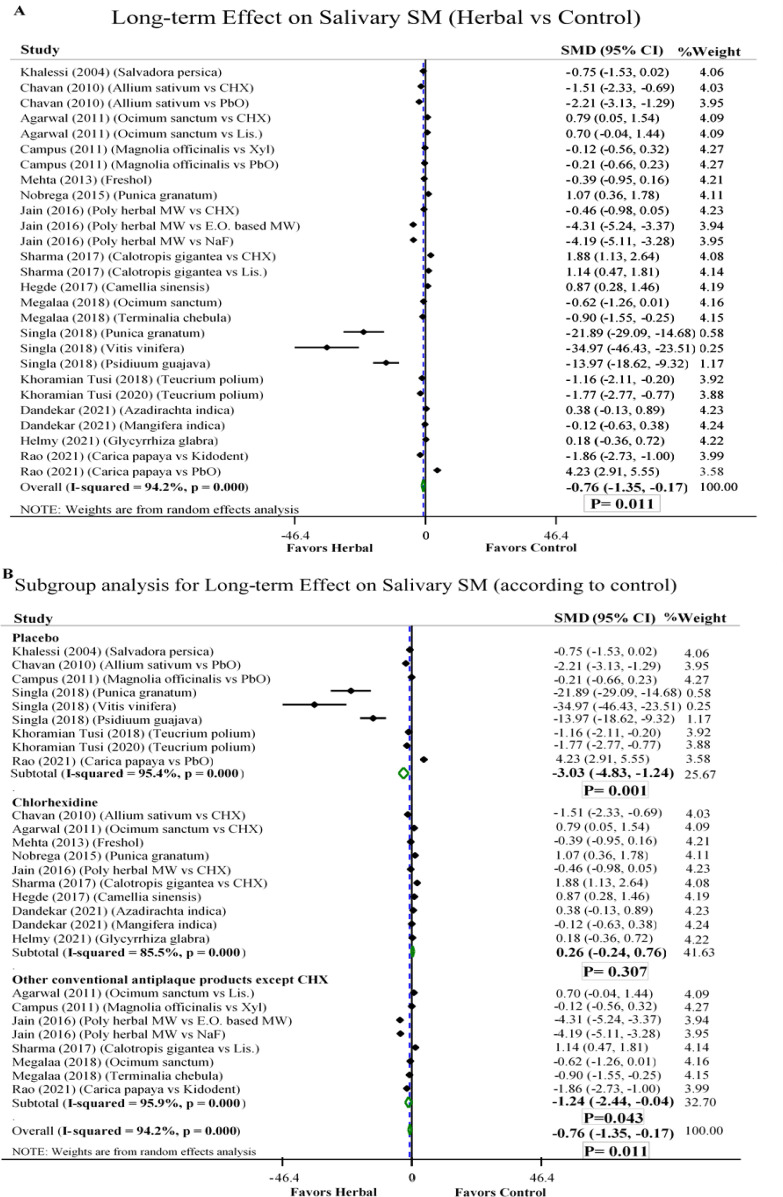
Forest plot of comparison: aqueous and alcoholic herbal extract vs A) all controls B) subgroups (Placebo, Chlorhexidine, other conventional antiplaque products); SM= *Streptococcus mutans*; CHX= Chlorhexidine; PbO= Placebo; E.O. = Essential oil; Lis. = Listerine; Xyl= Xylitol; NaF= sodium fluoride

Sixteen trials investigated long-term use of herbal products with a low risk of publication bias (Agarwal and Nagesh, 2011; Campus et al., 2011; Chavan et al., 2010; Dandekar and Winnier, 2021; Hegde and Kamath, 2017; Helmy et al., 2021; Jain and Jain, 2016; Khalessi et al., 2004; Khoramian Tusi et al., 2018; Khoramian Tusi et al., 2020; Megalaa et al., 2018; Mehta et al., 2013; Nobrega et al., 2015; Rao et al., 2021; Sharma et al., 2017; Singla et al., 2018). Overall, aqueous and alcoholic herbal extracts were significantly superior in reduction of SM count despite high heterogeneity among the studies [SMD -0.76, 95% CI (-1.35 to -0.17)] (Figure 3a). Nevertheless, the subgroup analysis yielded no significant difference between herbal products and chlorhexidine [SMD 0.26, 95% CI (-0.24 to 0.76)] (Figure 3b). 

Among 10 trials that used chlorhexidine, only one RCT showed significant superiority of a herbal extract (garlic) in reduction of SM compared to chlorhexidine, which was extremely different in demographic characteristics of participants from all the others as researchers enrolled dental students (Chavan et al., 2010). Two studies compared herbal products vs sodium fluoride and results significantly favored herbal products (Jain and Jain, 2016; Megalaa et al., 2018). However, two other trials favored Listerine ® over herbal products (Agarwal and Nagesh, 2011; Sharma et al., 2017).

**Long-term effect on SM level of plaque:**

**Figure 4:**
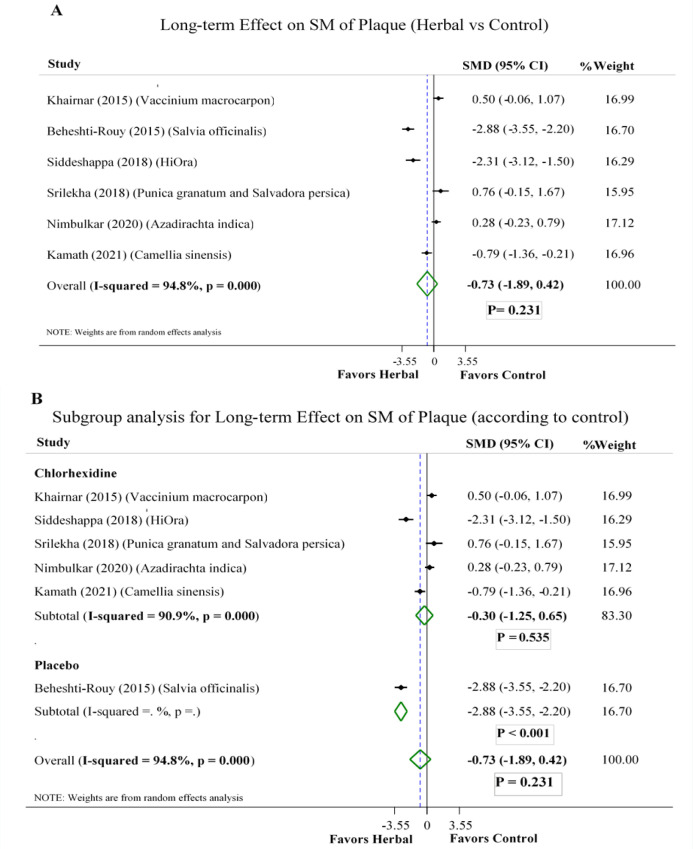
Forest plot of comparison: aqueous and alcoholic herbal extracts vs A) all controls B) subgroups (Placebo, chlorhexidine); SM = Streptococcus mutans

Pooled results of six studies (Beheshti-Rouy et al., 2015; Kamath et al., 2021; Khairnar et al., 2015; Nimbulkar et al., 2020; Siddeshappa et al., 2018; Srilekha and Prabakar, 2018) indicated a non-significant effect for herbal extracts over chlorhexidine in reducing SM of plaque [SMD -0.30, 95% CI (-1.25 to 0.65)] (Figure 4a, b).

The association between SM count of supra gingival plaque and dental caries is stronger in children than adults (Bhaumik et al., 2021); however, two trials enrolled children among the studies (Beheshti-Rouy et al., 2015; Kamath et al., 2021). 

**Adverse effects**

Jain et al. (2013) and Dandekar et al. (2021) suggested herbal mouthwash (*Glycyrrhiza glabra*, *Azadirachta indica*, and *Mangifera indica*) as a better-tasting alternative for chlorhexidine since it was more tolerable to children. Kamath et al. (2021) mentioned no lingering after taste for green tea (*Camellia sinensis*), whereas Agarwal et al. (2011), Kerdar et al. (2019), Khoramian Tusi et al. (2020) and Mishra et al. (2019) received complaints about bitter taste of herbal products (*Ocimum sanctum*, *Teucrium polium*, *Scrophularia striata*, and *Vitis vinifera*) which could be explained by their employed concentration. Nobrega et al. (2015) and Srinagesh et al. (2012) observed taste disturbance, lingering after taste and tooth staining as chlorhexidine side effects among participants. 

**Quality assessment**

Twenty-one trials with high risk of bias, 11 with an unclear risk and 25 with low risk were included in our systematic review (Table 2). The overall quality of evidence was considered low since inconsistency was regarded serious (Table 3).

## Discussion

Despite the preventable nature of untreated dental caries and recent decrease in their occurrence in high-income countries, they are still the most common health problem with a global burden similar to that of 30 years ago. This could be explained with unequal socioeconomic pattern and hence growing prevalence in disadvantaged communities (Kassebaum et al., 2017; Peres et al., 2019). Herein, we aimed to realize whether aqueous and alcoholic herbal extracts harbor equivalent efficacy as chemical antibacterial oral care agents in order to meet the needs of large population especially in low- and middle-income countries through the present meta-analysis.

Meta-analysis done based on immediate and long-term effects indicated that herbal products are generally superior to non-herbals in terms of salivary SM reduction. However, herbal products did not show significant difference in antibacterial potency compared to chlorhexidine (as the gold standard). Considering adverse effects of chlorhexidine (e.g. taste disturbance, dry mouth or xerostomia, tooth staining, antibacterial resistance, etc.), herbal products with minimal side effects and high acceptance level demonstrate potential as antibacterial oral care products (Brookes et al., 2020; Moshrefi, 2002). During our review of included studies, we found 3 trials using Listerine ® which did not favor herbal products (*Ocimum sanctum*, *Calotropis gigantea* and *Scrophularia striata*) in reduction of salivary SM over Listerine ® (Agarwal and Nagesh, 2011; Kerdar et al., 2019; Sharma et al., 2017). On the contrary, we observed substantial evidence on SM reduction by herbal products compared to sodium fluoride within 3 trials (Jain and Jain, 2016; Jauhari et al., 2015; Megalaa et al., 2018). All these results were strongly affected by heterogeneity.

 Janakiram et al. (2020) claimed no significant difference in reduction of Plaque index (PI) and Gingival index (GI) between herbal mouthwashes and chlorhexidine after 4 weeks of consumption. Meanwhile, chlorhexidine favored herbal mouthwash in reduction of PI and GI at 12 weeks. These results are somehow consistent with our study since the consumption period was not over 4 weeks in the studies included in our meta-analysis. Yet, this highlights the importance of prolonged consumptions. However, as Janakiram et al. (2020) focused on PI and GI, which their changes were found to be associated with SM count less than other bacterial species (Schaeken et al., 1987), compatibility of their review with ours should be considered with cautious. Despite stronger association between SM count of supra gingival plaque and dental caries in children than adults (Bhaumik et al., 2021), Janakiram et al. (2020) excluded children whom have great value for our study.

Our findings are in line with results reported by Jacob et al. (2018) showing no higher antibacterial potency for herbal products compared to chlorhexidine. Nonetheless, due to high heterogeneity and low quality of evidence we could not assure the substitution of chlorhexidine with the present knowledge.

 Furgium do Santo Cardosn et al. (2021) investigated RCTs focusing on periodontal and gingival indices and revealed *Camellia sinensis* as the most common herb for treatment of biofilm-associated pathologies which was employed by the studies included in the present review. But the studies included in the present review provided controversial results in terms of superiority of *Camellia sinensis* over conventional products (Hegde and Kamath, 2017; Kamath et al., 2021; Tehrani et al., 2011). Furthermore, Furgium do Santo Cardosn et al. (2021) observed that *Azadirachta indica* similarly affects periodontal indices compared to chlorhexidine. We also found 4 RCTs using *Azadirachta indica* throughout our review which suggested that *Azadirachta indica* and chlorhexidine had comparable potency on bacterial reduction (Botelho et al., 2008; Dandekar and Winnier, 2021; Nimbulkar et al., 2020; Pai et al., 2004; Patil et al., 2010; Selvaraj et al., 2020).

Our results are compatible with those published by Karygianni et al. (2015) who investigated the effect of medicinal plants on multispecies oral biofilm across laboratory studies and subsequently suggested them as supplement.

Although the presence of diverse molecules and active ingredients with various mechanisms of action in herbal products brings less bacterial resistance, broad-spectrum of action may occur which may lead to nonspecific unknown impacts on our body (Ferrazzano et al., 2011a; Furquim dos Santos Cardoso et al., 2021; Ribeiro et al., 2018). We tried to alleviate the severity of this issue by focusing on herbal extracts that are prepared with specified solvents (water and alcohol), as a result controlling the diversity of bioactive compounds. 

In addition, there was a lack of consistency in the intervention modality (e.g. frequency and duration of consumption, various dilutions of sample and incubation periods, etc.) and extensive difference in characteristics of participants (in terms of age, socioeconomic level, caries risk and oral hygiene). These all caused difficulties in analysis and interpretation of data. For instance, at least 48 hours of incubation period is required to accurately detect SM colonies (Wan et al., 2002). As a result, the diversity in incubation period can make data analysis challenging. 

According to a review by Bhaumik et al. (2021), there is a stronger association between SM count of plaque samples and caries in children than adults. Nevertheless, only within two trials, the plaque sample was collected from children. Kamath et al. (2021) reported a significant superiority of herbal product (*Camellia sinensis*) over chlorhexidine in children.

The substantial heterogeneity identified in our analysis is of potential concern. It may be attributed to the variety of plants that were used to prepare herbal products among the studies included in our review which leads to serious discrepancy. Unfortunately, there was a lack of evidence on side-effects in some of the included studies. All the above makes it difficult to draw specific conclusion.

There is a need to develop a standard uniform methodology (e.g. proposing unified bacterial counting methods and unified protocols for duration, quantity and frequency of daily consumption, unifying participants’ characterization, etc.) to provide precise information and gain more realistic view on the efficacy of herbal extracts. Besides, the effectiveness is better to be compared with chlorhexidine and be assessed in high-caries-risk participants to avoid overestimation of the effectiveness.

In light of possible lack of information on side-effects, further studies should address this issue by evaluating the safety of clinical usage at prolonged periods of time. Moreover, future studies are desired to measure the International Caries Detection and Assessment System (ICDAS) throughout an extended period of time since it is a better reflection of caries control capability. Reduction of heterogeneity could be sought through focusing on a single type of medicinal plant and its effectiveness in future meta-analyses.

 Aqueous and alcoholic herbal extracts-containing products seem to have more advantages in terms of effectiveness and safety over conventional non-herbal products; however, their effectiveness is not significantly greater than chlorhexidine. Concerning high level of heterogeneity, these results should be interpreted with caution. There is insufficient evidence, despite some low-quality evidence, to reach a definite conclusion recommend a substitution of conventional standard oral care products. Obviously, future high-quality trials are required to assert the safety and effectiveness of antibacterial herbal care products for preventing dental caries. 

**Table 1:** Search strategy applied for each database

**Bibliographic databases** ** (Primary sources)**	**Search strategy (Descriptors and boolean operators)**
**Medline via PubMed**	((aqueous extract) OR (alcoholic extract) OR ((herbal extract) OR ("Herbal Medicine"[Mesh])) OR (medicinal plant) OR (Chinese plant) OR (Indian plant) OR (Persian plant) OR (Iranian plant)) AND (dental OR oral OR teeth OR tooth) AND ((dental Decay) OR (anti caries agent) OR ((Streptococcus mutans) OR ("Streptococcus mutans"[Mesh])) OR ((dental caries) OR ("Dental Caries"[Mesh])) OR ((dental plaque) OR ("Dental Plaque"[Mesh])) OR ((dental OR oral OR teeth OR tooth) AND biofilm))
**Others:** **Scopus** **Web of Science** **Cochrane Library**	TITLE-ABS-KEY (((aqueous extract) OR (alcoholic extract) OR (herbal extract) OR (medicinal plant) OR (Chinese plant) OR (Indian plant) OR (Persian plant) OR (Iranian plant)) AND ((dental Decay) OR (anti caries agent) OR (Streptococcus mutans) OR (dental caries) OR (dental plaque) OR ((dental OR oral OR teeth OR tooth) AND biofilm))) ((aqueous extract) OR (alcoholic extract) OR (herbal extract) OR (medicinal plant) OR (Chinese plant) OR (Indian plant) OR (Persian plant) OR (Iranian plant)) AND ((dental Decay) OR (anti caries agent) OR (Streptococcus mutans) OR (dental caries) OR (dental plaque) OR ((dental OR oral OR teeth OR tooth) AND biofilm))
**Hand search**	Manual searches according to the reference lists of the articles

**Table 2:** General characteristics of the included studies; A: mouthwash; B: toothpaste; C: gel; D: gum; E: lollipop (classified as 1: studies on salivary sample, 2: studies on plaque sample)

Author (year)	Participants	Intervention group	Control group	Duration	Outcome	Risk of bias
** A1. Mouthwash (Salivary sample)**						
**Agarwal and Nagesh (2011)**	45 school children (14-15yr) with cavitated active carious lesion≥1 and salivary S.M. count above 10^5 ^CFU/ml	*Ocimum sanctum *(4%) (Tulsi)	Chlorhexidine (0.2%)	7 days	Significant superiority of chlorhexidine	Low risk
			Listerine ®		No significant difference	
**Al-Dabbagh et al. (2016)**	40 secondary school students (16-18yr) with carious teeth	*Salvadora persica* (Miswak^TM^)	Normal Saline	Immediate	Significant superiority of Miswak	High risk
				2 weeks	No significant difference	
**Al-Ezzi et al. (2018)**	30 dental students(21-23yr) with dental caries≥1	*Camellia sinensis* (Green tea)	Distilled water	Immediate	Not specified	Low risk
		Black tea			Not specified	
**Bajaj and Tandon (2011)**	1431 students(8-12yr)	Triphala (0.6%)	Chlorhexidine (0.1%)	9 months	Not specified	Some concerns
**Bhat et al. (2012)**	30 dental students(18-25yr) without caries experience	*Salvadora persica *(50%)	Normal Saline	Immediate	Significant superiority of *Salvadora persica*	High risk
**Bhat et al. (2017)**	20 children(8-14yr) From a residential school with good general health and a minimum of 12 gradable teeth	*Mangifera indica* (mango)	Chlorhexidine	Immediate	Significant superiority of chlorhexidine	High risk
				5 days	Significant superiority of chlorhexidine	
**Botelho et al. (2008)**	54 participants(17-65yr) gathered from slum community, high caries risk, natural teeth≥10, PI≥1.05, GI≥1.0	*Azadirachta indica* (neem) (25%)	Chlorhexidine (0.12%)	7 days	No significant difference	High risk
**Chandrashekar et al. (2019a)**	34 participants(18-30yr) recruited from a hostel with minimum 20 natural permanent teeth	poly herbal 1% (*Acacia nilotica*, *Murraya koenigii* Linn Sprengel, Eucalyptus, and *Psidium guajava*)	Chlorhexidine (0.12%)	15 days	Significant superiority of poly herbal	Low risk
			Distilled water		Significant superiority of poly herbal	
**Chavan et al. (2010)**	45 dental students(18-25yr)	*Allium sativum* (garlic) (3%)	Chlorhexidine (0.12%)	7 days	Significant superiority of garlic	Low risk
			Placebo		Significant superiority of garlic	
**Dandekar and Winnier (2021)**	90 Children(8-13yr) from a residential school with 3≤dmft/DMFT≤6	*Azadirachta indica* (%25)	Chlorhexidine (0.12%)	7 days	No significant difference	Low risk
		*Mangifera indica* (mango) (%25)			No significant difference	
**Ferrazzano et al. (2011b)**	66 dental patients (12-18yr) with good general health	*Camellia sinensis* (Green tea)	Distilled water	7 days	Significant superiority of *Camellia sinensis*	Some concerns
**Ferrazzano et al. (2015)**	44 dental patients(12-18yr) with good general health	*Plantago lanceolata*	Placebo	7 days	Significant superiority of *Plantago lanceolata*	High risk
**Hegde and Kamath (2017)**	75 children(8-12yr) with DMFT≥4	*Camellia sinensis* (Green tea) (5%)	Chlorhexidine (0.12%)	2 weeks	Significant superiority of chlorhexidine	Low risk
**Helmy et al. (2021)**	52 patients(18-55yr) with high caries risk and severe or active periodontal disease	*Glycyrrhiza glabra* (liquorice)	Chlorhexidine	Immediate	No significant difference	Low risk
				7 days	No significant difference	
**Jain et al.** **(2013)**	60children(7-14yr) with poor oral hygiene, DMFS & dmfs≥5	*Glycyrrhiza glabra *(liquorice)	Chlorhexidine (0.156%)	Immediate	Significant superiority of liquorice	Low risk
**Jain and Jain** **(2016)**	120 school children(15-17yr) with 3≤DMFT≤6	poly herbal (garlic, aqueous gooseberry, ginger)	Chlorhexidine (0.2%)	Immediate	No significant difference	Low risk
				7 days	No significant difference	
			Essential oil	Immediate	Significant superiority of poly herbal	
				7 days	Significant superiority of poly herbal	
			Sodium fluoride	Immediate	No significant difference	
				7 days	Significant superiority of poly herbal	
**Jauhari et al. (2015)**	52 healthy children(6-12yr)	*Salvadora persica*	Sodium Fluoride	2 weeks	No significant difference	High risk
			Distilled water		Significant superiority of *Salvadora persica*	
**Kerdar et al. (2019)**	50 participants (20-50yr) with mild to moderate chronic periodontitis	*Scrophularia striata*	Listerine ®	4 weeks	Not specified	High risk
**Khalessi et al. (2004)**	28 dental students(18-42yr)	*Salvadora persica* (Persica^TM^)	Placebo	3 weeks	Significant superiority of *Salvadora persica*	Low risk
**Khoramian Tusi et al. (2020)**	22 dental students(18-25yr) with no active caries and PI≤20%	*Teucrium polium* (0.2%)	Placebo	2 weeks	Significant superiority of *Teucrium polium*	Low risk
**Matsumoto et al. (2004)**	28 participants(19-29yr) with at least 24 teeth, 1<DMFT<13	Cacao bean (in 1% ethanol)	Placebo (1% ethanol)	4 days	No significant difference	Some concerns
**Megalaa et al. (2018)**	60 children(6-12yr) with high cries risk	*Ocimum sanctum * (2.5%)	Sodium Fluoride (0.05%)	Immediate	Significant superiority of *Ocimum sanctum*	Some concerns
				7 days		
		Terminalia chebula (4%)		Immediate	Significant superiority of *Terminalia chebula*	
				7 days		
**Mehta et al. (2013)**	55 children(8-14yr) with good general health and gradable teeth≥12	Freshol (staphysagria, chamomilla, echinacea, plantago, ocimum, cistus)	Chlorhexidine	10 days	Significant superiority of Freshol	High risk
**Mishra et al. (2019)**	80 children (8-15yr) recruited from a residential premise, DMFT/dmft>4	*Punica granatum*	Chlorhexidine (0.2%)	15 days	No significant difference	High risk
		*Terminalia chebula*				
		*Vitis vinifera* (grape)				
**Nayak et al. (2010)**	30 participants (20-25yr)	*Terminalia chebula* (10%)	Distilled water	Immediate	Significant superiority of *Terminalia chebula*	Some concerns
**Nayak et al. (2012)**	60 children(12-15yr) 3≤DMFT≤6, GI: moderate (Loe and Silness), PI: fair (Silness and Loe)	*Terminalia chebula* (2.5%)	Placebo	Immediate	Significant superiority of *Terminalia chebula*	Low risk
**Nobrega et al. (2015)**	35 students(9-12yr) with good general health, Simplified Oral Hygiene Index≥1.6, Teeth≥20	*Punica granatum* (6.25%)	Chlorhexidine (0.12%)	2 weeks	Significant superiority of chlorhexidine	Low risk
**Oznurhan et al. (2019)**	90 children(10-13yr) with simple gingivitis	*Glycyrrhiza glabra* (liquorice)	Chlorhexidine	Immediate	No significant difference	Low risk
			Normal Saline		No significant difference	
**Pathi et al. (2021)**	45 children(7-14yr) From an orphanage, high caries risk, Salivary S.M.≥ 10^5^ CFU/ml	*Glycyrrhiza glabra* (liquorice)	Chlorhexidine (0.12%)	Immediate	Significant superiority of liquorice	Low risk
**Pinni et al. (2018)**	30 children(8-12yr) with DMFT=4	*Punica granatum* (50 mg/ml)	Chlorhexidine (0.2%)	Immediate	No significant difference	Some concerns
			Distilled water		Significant superiority of *Punica granatum*	
**Rao et al. (2021)**	60 school children(8-12yr)	*Carica papaya*	Kidodent (Sodium monofluorophosphate, Triclosan, Xylitol)	15 days	Significant superiority of *Carica papaya*	High risk
			Distilled water		Significant superiority of Distilled water	
**Sharma et al. (2017)**	60 children(14-15yr) from different schools, cavitated active carious lesion≥1, Salivary S.M. count≥10^5^ CFU/ml	*Calotropis gigantea* (2.5%)	Chlorhexidine (0.2%)	7 days	Significant superiority of chlorhexidine	High risk
			Listerine ®		Significant superiority of Listerine	
**Siddeshappa et al. (2018)**	40 participants (20-50yr) with mild-to-moderate gingivitis, teeth≥20, without untreated caries	HiOra* (*Salvadora persica*, *Terminalia bellirica*, *Piper betle)*	Chlorhexidine	3 weeks	Significant superiority of HiOra	Low risk
**Singla et al. (2018)**	40 Children (8-10yr) from a local boarding school with high caries risk	*Punica granatum* (50%)	Distilled water	7 days	Significant superiority of *Punica granatum*	Some concerns
		*Vitis vinifera* (Grape seed) (12.5%)			Significant superiority of *Vitis vinifera*	
		*Psidium guajava* (Guava seed) (25%)			Significant superiority of *Psidium guajava*	
**Srikanth et al. (2008)**	32 Students (10-14yr) from a residential school, refrained from other routine oral practices during the study	Cocoa bean husk	Placebo	4 days	Significant superiority of cocoa	High risk
**Srinagesh et al. (2012)**	60 undergraduate students (18-25yr) with a frank carious lesion and/or active incipient caries	Triphala (0.6%)	Chlorhexidine (0.2%)	7 days	No significant difference	Some concerns
**Tehrani et al. (2011)**	60 Children (8-12yr) from different schools, without untreated active caries, gingivitis or periodontal diseases	*Camellia sinensis* (Green tea) (0.5%)	Sodium Fluoride (0.5%)	2 weeks	No significant difference	Low risk
**Umar et al. (2016)**	50 participants (15-25yr) without cavity, probing depth<4^mm^, saliva flow rate: 0.5 ml/min	*Punica granatum* (300mg/ml)	Chlorhexidine (0.2%)	Immediate	Significant superiority of *Punica granatum*	High risk
**Velmurugan et al.** **(2013)**	45 participant with High caries risk	*Terminalia chebula* (20%)	Chlorhexidine (0.2%)	Immediate	No significant difference	Some concerns
		*Phyllanthus emblica * (20%)			Significant superiority of Phyllanthus emblica	
**Yadav et al. (2017)**	45dental students(18-22yr),1.2≤DMFT≤2.6	*Coffea canephora *	Chlorhexidine (0.2%)	2 weeks	No significant difference	High risk
			sterile water		Significant superiority of coffee	
** A2. Mouthwash ( Plaque sample)**						
**Beheshti-Rouy et al. (2015)**	Plaque sample of 70 girls (11-14yr) from a dormitory	*Salvia officinalis* 5% (Sage)	Normal Saline	3 weeks	Significant superiority of Salvia officinalis	Low risk
**Chandrashekar et al. (2019b)**	30 participants(18-30yr) From a free hostel, natural permanent teeth≥20, refrained from other oral hygiene practices during the study	poly herbal 1% (*Acacia nilotica*, *Murraya koenigii* Linn Sprengel, Eucalyptus hybrid, and *Psidium guajava*)	Chlorhexidine (0.12%)	4 days	Not specified	Low risk
			Distilled water			
**Kamath et al. (2021)**	50 children(8-12yr) dmft≥4	*Camellia sinensis *%0.5 (Green tea)	Chlorhexidine 0.12%	2 weeks	Significant superiority of Camellia sinensis	Low risk
**Khairnar et al. (2015)**	50 participants(18-20yr)	*Vaccinium macrocarpon* (Cranberry)	Chlorhexidine (0.2%)	2 weeks	No significant difference	Some concerns
**Preethy and Somasundaram (2021)**	50 children(8-10yr) with good general health and minimal of 3-4 active carious lesions	*Vaccinium macrocarpon* 0.6% (Cranberry)	Chlorhexidine (0.2%)	2 weeks	No significant difference	High risk
**Shinada et al. (2007)**	26 men(20-38yr) without periodontitis	*Humulus lupulus *(hops)	Placebo	3 days	Significant superiority of *Humulus lupulus*	Low risk
**Srilekha and Prabakar (2018)**	20 dental students(18-25yr), PI: good to fair, mild to moderate gingivitis, habit of tooth brushing twice daily	*Punica granatum* (+*Salvadora persica* & fluoride)	Chlorhexidine	7 days	No significant difference	Some concerns
**Toothpaste ( Salivary sample)**						
**Al-Dabbagh et al. (2016)**	40 school Students(16-18yr) with carious teeth≥2	*Salvadora* *Persica* (Siwak.F)	ordinary (Doctor Toothpaste)	Immediate	Significant superiority of *Salvadora* *Persica*	High risk
				2 weeks		
**Patil et al. (2010)**	100 preschool children (4-6yr) with dmft=0	Himalaya (*Azadirachta indica)*	Cheerio gel (fluoride 485PPM)	150 days	No significant difference	High risk
**Selvaraj et al. ** **(2020)**	60 participants(18-30yr)	Babool (*Azadirachta indica*)	PerioBiotic	60 days	Significant superiority of *Azadirachta indica*	High risk
** C1. Gel (Salivary sample)**						
**Kumar et al. (2020)**	30 children(6-12yr) with decayed teeth≥4	*Glycyrrhiza glabra* (liquorice)	Chlorhexidine (0.2%)	Immediate	Significant superiority of liquorice	High risk
**Pai et al. (2004)**	36 participants	*Azadirachta indica* (25 mg/g)	Chlorhexidine 0.12%	6 weeks	Significant superiority of *Azadirachta indica*	High risk
			Placebo			
**Sajadi et al. (2021)**	90 preschool children(4-6yr) without active and severe periodontal diseases	*Thymus vulgaris* (thyme)(5%)	Chlorhexidine (0.2%)	Immediate	No significant difference	Some concerns
				7 days	No significant difference	
		*Matricaria chamomile* (5%)		Immediate	Significant superiority of chlorhexidine	
				7 days	No significant difference	
** C2. Gel ( Plaque sample)**						
**Nimbulkar et al. (2020)**	60 teachers(20-30yr)	*Azadirachta indica* (2.5%)	Chlorhexidine (0.2%)	90 days	No significant difference	Low risk
** D. Gum ( Salivary sample)**						
**Campus et al. (2011)**	120 adults(18-30yr) with high caries risk (SM≥10^5^ CFU/ml, BOP>25%, 1<carious lesion<4)	*Magnolia officinalis* 0.17% + 30% Xylitol	Xylitol (30%)	30 days	Significant superiority of Magnolia officinalis	Low risk
			Placebo			
**Gao et al. (2018)**	20 healthy young adults(20-35yr)	*Phyllanthus emblica* 10%	Placebo	Immediate	Significant superiority of Phyllanthus emblica	High risk
**Khoramian Tusi et al. (2018)**	20 dental students(20-30yr) with no active caries	*Teucrium polium*	Placebo	21 days	Significant superiority of Teucrium polium	Low risk
** E. Lollipop ( Salivary sample)**						
**Almaz et al. (2017)**	108 healthy children(5-11yr) from both caries-free and high caries risk categories	*Glycyrrhiza glabra* (liquorice)	Placebo	10 days	No significant difference	Low risk

**Table 3:** Quality assessment using GRADE approach

***Certainty assessment*** **No. of studies**	**Risk of bias**	**Inconsistency**	**Indirectness**	**Imprecision**	**Publication bias**	**Certainty**
Immediate Effect on Salivary SM^1^: 7	not serious	serious	not serious	not serious	not serious	⊕⊕ Low
Long-term effect on Salivary SM: 16	not serious	serious	not serious	not serious	not serious	⊕⊕ Low
Long-term effect on SM of Plaque: 6	not serious	serious	not serious	not serious	not serious	⊕⊕ Low

1: SM= *Streptococcus mutans*
